# Novel TrxR1 Inhibitors Show Potential for Glioma Treatment by Suppressing the Invasion and Sensitizing Glioma Cells to Chemotherapy

**DOI:** 10.3389/fmolb.2020.586146

**Published:** 2020-10-06

**Authors:** Mirna Jovanović, Miodrag Dragoj, Daniil Zhukovsky, Dmitry Dar’in, Mikhail Krasavin, Milica Pešić, Ana Podolski-Renić

**Affiliations:** ^1^Department of Neurobiology, Institute for Biological Research “Siniša Stanković” – National Institute of Republic of Serbia, University of Belgrade, Belgrade, Serbia; ^2^Institute of Chemistry, Saint Petersburg State University, Russian Federation, Saint Petersburg, Russia

**Keywords:** glioma, multidrug resistance, thioredoxin reductase 1, oxidative stress, temozolomide, invasion

## Abstract

Currently, available glioblastoma (GBM) treatment remains ineffective, with relapse after initial response and low survival rate of GBM patients. The reasons behind limited capacities for GBM treatment are high tumor heterogeneity, invasiveness, and occurrence of drug resistance. Therefore, developing novel therapeutic strategies is of utmost importance. Thioredoxin reductase (TrxR) is a novel, promising target due to its overexpression in many cancer types and important role in cancer progression. Previous research on Ugi-type Michael acceptors–inhibitors of TrxR showed desirable anticancer properties, with significant selectivity toward cancer cells. Herein, two TrxR inhibitors, **5** and **6**, underwent in-depth study on multidrug-resistant (MDR) glioma cell lines. Besides the antioxidative effects, **5** and **6** induced cell death, decreased cell proliferation, and suppressed invasion and migration of glioma cells. Both compounds showed a synergistic effect in combination with temozolomide (TMZ), a first-line chemotherapeutic for GBM treatment. Moreover, **5** and **6** affected activity of P-glycoprotein extrusion pump that could be found in cancer cells and in the blood–brain barrier (BBB), thus showing potential for suppressing MDR phenotype in cancer cells and evading BBB. In conclusion, investigated TrxR inhibitors are effective anticancer compounds, acting through inhibition of the thioredoxin system and perturbation of antioxidative defense systems of glioma cells. They are suitable for combining with other chemotherapeutics, able to surpass the BBB and overcome MDR. Thus, our findings suggest further exploration of Ugi-type Michael acceptors–TrxR inhibitors’ potential as an adjuvant therapy for GBM treatment.

## Introduction

Glioblastoma (GBM) is the most common and aggressive malignant brain tumor in adults, with unfavorable prognosis (Stupp et al., 2005). Only a few chemotherapeutics are used for GBM treatment due to the very low biodistribution of most anticancer drugs in the brain as a consequence of high P-glycoprotein (P-gp) expression in the blood–brain barrier (BBB) (Da Ros et al., 2018). The current standard procedure for GBM treatment is surgical resection, followed by radiotherapy with concomitant temozolomide (TMZ) chemotherapy, an agent that can cross BBB. However, tumor recurrence occurs in high frequency, and the median survival rate of GBM patients is 15–18 months (Ostrom et al., 2016). The reasons for ineffective GBM treatment are high heterogeneity of tumor cells, aggressive invasiveness, and resistance to therapy (Salazar-Ramiro et al., 2016).

Multidrug resistance (MDR), as a serious obstacle for efficient cancer treatment, represents the leading cause of cancer treatment failure (Holohan et al., 2013). The acquisition of MDR involves several complex mechanisms and occurs when cancer cells develop resistance to structurally and mechanistically unrelated anticancer drugs (Kachalaki et al., 2016). The concept of MDR is commonly associated with overexpression of P-gp, a membrane transporter that extrudes a wide range of anticancer drugs out of the cancer cells as well as out of the cells present in the BBB (Fletcher et al., 2016). One of the features of MDR cancer cells is the enhanced activity of the antioxidant system due to increased levels of reactive oxygen and nitrogen species (RONS). These reactive species can further activate redox-sensitive transcription factors that upregulate P-gp (Scotto, 2003), thus providing additional protection against chemotherapy.

Genetic mutations associated with GBM pathogenesis are accompanied by aberrations in metabolism, mitochondrial dysfunction, and increased oxidative stress (Salazar-Ramiro et al., 2016). The elevated RONS level is a common feature of cancer cells, leading to abnormal cell growth, metastasis, evasion of cell death, and resistance to therapy (Moloney and Cotter, 2018). To adapt to oxidative stress, cancer cells activate mechanisms of defense, involving a number of free radical scavengers (Leone et al., 2017). The main systems included in antioxidant defense are redox-regulating glutathione (GSH) and thioredoxin (Trx) systems, as well as other enzymes neutralizing reactive species inside of the cell, such as catalase (CAT) and superoxide dismutase (SOD).

GSH is a small peptide with a major role in antioxidant defense, either by direct interaction with RONS or by acting as a cofactor for GSH peroxidases (GPxs) and GSH-S transferases (GSTs). GPx scavenges organic and inorganic peroxides inside of the cell (Lushchak, 2012), oxidizing GSH to GSSG. Oxidized GSH is reduced by NADPH-dependent GSH reductase (GR). GSTs also catalyze the conjunction of xenobiotics with GSH, thus neutralizing the potentially harmful molecules (Lushchak, 2012). SOD transforms superoxide radicals into molecular oxygen and hydrogen peroxide. Two main types of SOD are cytoplasmic Cu/ZnSOD and mitochondrial MnSOD, which mainly remove free radicals as products of electron-transport chain activity (Birben et al., 2012). CAT mediates decomposition of hydrogen peroxide, partially overlapping in substrate specificity and function with GPx (Birben et al., 2012).

One of the major systems responsible for maintenance of redox equilibrium inside of the cell is the Trx system, comprising Trx, Trx reductase (TrxR), and NADPH. Trx reduces target proteins, while TrxR recovers Trx reduced state at the expense of NADPH as an electron donor. As redox regulators, Trx and TrxR participate in many cellular events, e.g., defense response to oxidative stress, growth, proliferation, and apoptosis (Karlenius and Tonissen, 2010). Trx has broad substrate specificity. Trx target proteins include peroxiredoxins (Lu and Holmgren, 2014), another protein reducing peroxides in cells, alongside with GPx and CAT. Trx has an important role in cell proliferation by regulating the activity of ribonucleotide reductase (RNR), a key enzyme involved in DNA replication (Lu and Holmgren, 2014). One of the target proteins of Trx is apoptosis signal-regulating kinase 1 (ASK1); reduced Trx keeps ASK1 inactive, thus inhibiting apoptosis. However, during oxidative stress, concentration of the reduced form of Trx decreases, and consequently, ASK1 activates apoptotic signaling pathways (Saitoh et al., 1998; Lu and Holmgren, 2014).

Accumulative evidence indicates the importance of Trx system in tumor progression and metastasis. A previous study demonstrated increased expression of TrxR in GBM patients (Kemerdere et al., 2013) that correlated with higher resistance to radiotherapy and chemotherapy (Leone et al., 2017). TrxR expression is higher in primary GBM and associates with poor prognosis (Erdi et al., 2018), as well as increased angiogenesis (Kaya et al., 2016). It has been established that some conventional chemotherapeutics (including carmustine, a drug used for GBM treatment) possess the capacity to inhibit TrxR (Cai et al., 2012). Their anticancer effect can, at least in part, be ascribed to the inhibition of TrxR. Auranofin, a well-known specific TrxR inhibitor approved for treating rheumatoid arthritis, has been investigated for cancer treatments (Li et al., 2016; Wang et al., 2017; Parrales et al., 2018). Lately, quite a few researches have been directed toward exploring the potential of novel inhibitors of Trx and TrxR for cancer therapy (Zhang et al., 2017; Mohammadi et al., 2019). Therefore, targeting the Trx system might be an attractive approach for GBM treatment.

Previously, we identified six new Ugi-type Michael acceptors (UMAs) (**1**, **2**, **3**, **4**, **5**, and **6**) that proved to be potent TrxR1 inhibitors (Jovanovic et al., 2019). Here, we report anticancer properties of six UMAs on human and rat MDR glioma cell lines and their sensitive counterparts (Podolski-Renic et al., 2011; Stojkovic et al., 2015). After the initial analysis of cytotoxic activity and potential to induce RONS, we identified two leading compounds (**5** and **6**) for further exploration of their mechanisms of action. Since human and rat MDR glioma cell lines possess different antioxidative capacities (Stankovic et al., 2015; Stojkovic et al., 2015), we aimed to compare their vulnerability to oxidative stress induced by TrxR1 inhibitors. Therefore, we further assessed their impact on the antioxidative defense system, mitochondrial membrane potential, cell death induction, and cell proliferation. We also investigated combined effects of **5** and **6** with TMZ in glioma cell lines as well as the potential to sensitize human MDR glioma cells to paclitaxel (PTX). Also, we explored the anti-invasive properties of TrxR1 inhibitors in glioma cell lines.

## Materials and Methods

### UMAs’ Structure

Compounds **1**–**6** were prepared via the Ugi reaction as described previously (Jovanovic et al., 2019). While all six compounds feature a Michael acceptor β-aroyl acrylic acid residue (shown in red), compound **6** (DVD-445) has it attached via an ester linkage while the rest of the set are β-aroyl acrylamides (Supplementary Figure 1).

### Cell Culture

Human glioblastoma cell lines U87 and U87-TxR were grown in Minimum Essential Medium (M2279-500ML, Sigma, United Kingdom) supplemented with 10% fetal bovine serum (F0804, Sigma-Aldrich, Germany), 2 mM glutamine (G7513-100ML, Sigma, United Kingdom), 5,000 U/ml penicillin, and 5 mg/ml streptomycin (cat. no. 15140122, Gibco^TM^, Thermo Fisher Scientific, United States). Rat glioma cell lines C6 and RC6 were grown in Dulbecco’s Modified Eagle Medium (cat. no. 01-056-1A, Biological Industries, United States), supplemented with 10% fetal bovine serum, 2 mM L-glutamine, 5,000 U/ml penicillin, and 5 mg/ml streptomycin. U87 and C6 cell lines were obtained from American Type Culture Collection (ATCC, Rockville, MD, United States) and the U87-TxR cell line was selected by continuous exposure to stepwise increasing concentrations of PTX from the U87 cell line, while the RC6 cell line was established from the C6 cell line after carmustine selective pressure (Podolski-Renic et al., 2011; Stojkovic et al., 2015). All cell lines were grown at 37°C in a humidified 5% CO_2_ atmosphere.

### MTT Assay

Cells grown in 75 cm^2^ tissue flasks were trypsinized, seeded into flat-bottomed adherent 96-well cell culture plates in appropriate medium (U87 and U87-TxR 4,000 cells per well, C6 and RC6 2,000 cells per well), and incubated overnight. The cells were exposed to increasing concentrations of the tested compounds for 72 h: **2** and **6** (1–50 μM), **3** and **4** (0.25–5 μM), **5** (1–25 μM), and **1** (0.25–5 μM) in rat glioma cells as well as **1** (1–50 μM) in human glioblastoma cells. The combined effects of **5** and **6** with TMZ (T2577, Sigma-Aldrich, Germany) were also studied. In simultaneous treatments that lasted 72 h in C6, U87, and U87-TxR cells, three concentrations of **5** (0.5, 1, and 1.5 μM) and three concentrations of compound **6** (1, 2.5, and 5 μM) were combined with increasing concentrations of TMZ (10, 25, and 50 μM), while in RC6 cells the same concentrations of **5** and **6** were combined with different concentrations of TMZ (100, 250, and 500 μM). In simultaneous treatments of **5** and **6** with PTX (T1912, Sigma-Aldrich, United States), three concentrations of **5** (0.25, 0.5, and 1 μM) and three concentrations of **7** (1, 2.5, and 5 μM) were combined with PTX (0.1–2.5 μM) in U87-TxR cell line. The metabolic activity of viable cells was measured by MTT assay (Mosmann, 1983). Briefly, medium containing 0.2 mg/ml of thiazolyl blue tetrazolium bromide (MTT, M2128, Sigma-Aldrich, Germany) was added to each well. After incubation (3 h, at 37°C in a 5% CO_2_), the MTT-containing medium was removed, and 200 μl of dimethyl sulfoxide was immediately added to each sample. Absorbance was measured at 570 nm on Multiskan Sky Microplate Spectrophotometer (Thermo Fisher Scientific, United States). The half-maximal inhibitory concentration (IC_50_) of each compound was calculated using GraphPad Prism^®^ 6.0 (GraphPad Software, Inc., United States).

### Median Effect Analysis

The nature of the interaction between TMZ and **5** or **6** was analyzed using CalcuSyn software (Biosoft, Cambridge, United Kingdom) that uses the combination index method, based on the multiple drug effect equation (Chou and Talalay, 1984). Three data points were used for every single drug in each designed experiment. The non-constant ratio combination was chosen to determine the effect of both drugs in combination. The results are presented in a fraction-affected combination index (CI) graph. The values of CI < 1 indicate synergism, a value of CI = 1 indicates an additive effect, while values of CI > 1 point to antagonism.

### RONS Production

Dihydrorhodamine 123 (DHR, D1054, Sigma-Aldrich, United States) fluorescent dye was used to assess RONS levels in U87, U87-TxR, C6, and RC6 cells. Levels of hydrogen peroxide and peroxynitrite anions, activating DHR (Jourd’heuil et al., 2001), were determined. Cells were plated and incubated overnight in six-well plates (100,000 cells per well). Then, cells were treated for 24 h with 1.5 μM **1**, 5 μM **2**, 0.5 μM **3**, 0.8 μM **4**, 2 μM **5**, and 8 μM **6** for rat glioma cells and 2 μM **1**, 15 μM **2**, 0.5 μM **3**, 0.6 μM **4**, 2 μM **5**, and 8 μM **6** for human glioblastoma cells. After treatments, adherent cells were harvested by trypsinization and incubated in a medium with 5 μM DHR for 30 min at 37°C in the dark. Cells were subsequently washed twice in PBS. DHR fluorescence was assessed in FL1 green channel. A minimum of 10,000 events was assayed for each sample. Fluorescence was measured on CyFlow Space flow cytometer (Partec, Münster, Germany), after which results were analyzed by Summit analysis software.

### RNA Extraction and Reverse Transcription Reaction

All cell lines were treated with 2 μM **5** and 8 μM **6** for 24 h prior to RNA isolation. Total RNA was isolated from cells with TRI Reagent^TM^ Solution (AM9738, Thermo Fisher Scientific, United States) according to the manufacturer’s instructions. RNA was quantified by spectrophotometry, and quality was determined by agarose gel electrophoresis. Reverse transcription was performed using a High-Capacity cDNA Reverse Transcription Kit (cat. no. 4374966, Applied Biosystems^TM^, Thermo Fisher Scientific, United States).

### Quantitative Real-Time PCR

To determine mRNA expression levels of *Trx*, *TrxR1*, *GPx1*, *GPx4*, *GST*π, *GR*, *MnSOD*, *CAT*, and *ACTB* in U87, U87-TxR, C6, and RC6 cells, quantitative real-time PCR (qPCR) was performed using specific primers (O’Driscoll et al., 1993; Larrea et al., 1998; Mansur et al., 1998; NicAmhlaoibh et al., 1999; Kamerbeek et al., 2007; Cha et al., 2009; Messaoudi et al., 2010; Paukert et al., 2011; Yagublu et al., 2011; Zhu et al., 2011; Vesentini et al., 2012; Miler et al., 2016; Stojkovic et al., 2016; Hemshekhar et al., 2017). Prepared cDNAs were amplified using Maxima SYBR Green/ROX qPCR Master Mix (K0222, Thermo Scientific, United States), on an ABI PRISM 7000 Sequence Detection System (Applied Biosystems, United States) according to manufacturer recommendations. Thermocycler conditions comprised an initial step at 50°C for 5 min, followed by a step at 95°C for 10 min and a subsequent two-step PCR program at 95°C for 15 s and 60°C for 60 s for 40 cycles. Each sample was tested in triplicate, and relative gene expression was analyzed by the 2^–Δ^ ^Δ^ ^Ct^ method (Livak and Schmittgen, 2001), Δ^Ct^ being the difference between Ct values of specific genes and the endogenous control (*ACTB*).

### Protein Expression Analysis

Expression subunit RRM1 of RNR, ASK1, and TrxR1 proteins was analyzed by flow cytometry. Sensitive and MDR cells were treated with 2 μM **5** or 8 μM **6**. After 24 h treatment, the cells were trypsinized, washed with PBS, and fixed in 4% paraformaldehyde for 10 min at 4°C. Samples were then permeabilized by ice-cold absolute methanol overnight at −20°C. After washing in PBS, cells were blocked for 1 h in 0.5% BSA in PBS. Cell pellet was than resuspended in primary antibodies, diluted in 0.5% BSA [1:50 RRM1 (ab137114, Abcam, United Kingdom), 1:200 ASK1 (ab45178, Abcam, United Kingdom), and 1:500 TrxR1 (ab124954, Abcam, United Kingdom), and incubated overnight at 4°C. Following incubation, samples were washed twice in 0.5% BSA, resuspended in fluorescently labeled anti-rabbit IgG secondary antibody (Alexa Fluor^®^ 488 Conjugated, #4412, Cell Signaling Technology, United States), diluted 1:1,000 in 0.5% BSA, and incubated for 1 h at room temperature in the dark. The fluorescence was measured in the FL1 channel on CyFlow Space flow cytometer (Partec, Münster, Germany) and analyzed by Summit analysis software. A minimum of 10,000 events was assayed per sample.

### Mitochondrial Membrane Potential Detection

JC-1 assay kit (cat. no. 551302, BD Biosciences, San Diego, CA, United States) was used for the detection of mitochondrial membrane potential. JC-1 is a cationic, lipophilic dye, exhibiting potential-dependent accumulation in mitochondria. In functional mitochondria, the dye aggregates fluorescing in red (FL2 channel of flow cytometer), while in the cytoplasm, it remains as monomers fluorescing in green (FL1 channel of flow cytometer). In the state of mitochondrial depolarization, JC-1 leaks out of the mitochondria into the cytoplasm, resulting in a decrease in the red/green fluorescence intensity ratio. Cells were plated and incubated overnight in six-well plates (100,000 cells per well) and then treated for 24 h with 2 μM **5** or 8 μM **6**. Carbonyl cyanide *m*-chlorophenyl hydrazine (CCCP, C2759, Sigma-Aldrich, Germany) was used as a positive control for mitochondrial membrane potential depolarization. According to the manufacturer’s instructions, following trypsinization, cells were incubated with a JC-1 reagent for 15 min at 37°C in a CO_2_ incubator. After incubation, cells were washed in 1 × Assay Buffer and resuspended in PBS before flow cytometric analysis. Both red and green fluorescence emissions were detected, and their ratio was analyzed on a CyFlow Space flow cytometer (Partec, Münster, Germany) (excitation λ = 488 nm, emission λ = 530 nm for FL1 channel and 585 nm for FL2 channel). A minimum of 10,000 events was assayed per sample. Results were analyzed by Summit analysis software.

### Cell Viability Assay

The percentages of viable, early apoptotic, late apoptotic, and necrotic cells were determined by annexin V/propidium iodide (AV/PI) labeling, using Annexin V-FITC Apoptosis Detection Kit (ab14085, Abcam, United Kingdom). All cell lines were seeded in adherent six-well plates (200,000 cells per well) and incubated overnight. Plated cells were treated with 2 μM **5** and 8 μM **6**. After 72 h treatment, total (attached and floating) cells were collected. The cell pellet was resuspended in 100 μl of binding buffer, containing AV and PI in a ratio of 1:1 (v/v). After the incubation period (10 min at room temperature in the dark), an additional 400 μl of binding buffer was added and AV/PI staining was analyzed within 1 h by flow cytometry. The fluorescence intensity was measured in green FL1 and red FL2 channel on CyFlow Space flow cytometer (Partec, Münster, Germany). In each sample, 20,000 cells were recorded, and the percentages of viable (AV− PI−), early apoptotic (AV + PI−), late apoptotic (AV + PI +), and necrotic (AV− PI +) cells were analyzed.

### Determination of Cell Proliferation

Cell proliferation was assessed by flow cytometry using carboxyfluorescein succinimidyl ester (CFSE, C34570, Invitrogen^TM^, Thermo Fisher Scientific, United States) dye (Kang et al., 2005). The fluorescence intensity of CFSE gradually declines during cell divisions, thereby enabling the assessment of cell proliferation rates in treated versus untreated cells (Lyons and Parish, 1994). U87 and U87-TxR cells were detached and stained with 5 μM CFSE for 15 min in the dark at 37°C in 5% CO_2_. After incubation, cells were washed three times in PBS and seeded on six-well plates. Cells were incubated on a plate for 2 h and treated with 2 μM of compound **5** and 8 μM of compound **6**. Following 72 h treatment, cells were trypsinized, washed twice, and resuspended in PBS. Green fluorescence emission was measured on the FL1 channel of CyFlow Space flow cytometer (Partec, Germany). A minimum of 20,000 events was assayed per sample. The results were analyzed in Summit analysis software.

### Rhodamine 123 Accumulation Assay

Accumulation of fluorescent P-gp substrate rhodamine 123 (Rho123, R8004, Sigma-Aldrich, Germany) was analyzed by flow cytometry. The intensity of emitted fluorescence is proportional to Rho123 accumulation inside the cell. The assay was performed with **5**, **6**, and a well-known P-gp inhibitor, tariquidar (TQ, kindly provided by Dr. Sven Rottenberg from The Netherlands Cancer Institute, Amsterdam), in U87-TxR cells. U87 cells were used as a positive control for Rho123 accumulation. U87-TxR cells were treated with 1 μM **5**, 5 μM **6**, and 50 nM TQ and incubated with 5 μM Rho123 for 30 min at 37°C in 5% CO_2_. At the end of the accumulation period, the cells were pelleted by centrifugation, washed with PBS, and placed in cold PBS. Accumulation of Rho123 was analyzed on CyFlow Space flow cytometer (Partec, Germany). The orange fluorescence of Rho123 was assessed in FL1 green channel at 530 nM. A minimum of 10,000 events was assayed per sample.

### Gelatin Degradation Analysis

Glioma cell lines were plated in six-well plates with glass coverslips, coated with Alexa Fluor 488-labeled gelatin (G13186, Gelatin from Pig Skin, Oregon Green^®^ 488 Conjugate, Life Technologies, Waltham, MA, United States). After 24 h of treatment with 2 μM **5** or 8 μM **6**, cells were fixed with 4% paraformaldehyde and co-stained with Hoechst 33342 and Actin Red 555 (R37112, Molecular Probes^TM^, Life Technologies, United States). Cells and degradation areas were analyzed under a Zeiss Axio Vert inverted fluorescent microscope (Carl Zeiss Foundation, Heidenheim, Germany). The volume of the dark area caused by the degradation of gelatin was measured in ImageJ software (1.48, Microsoft, Redmond, WA, United States) and normalized to the number of the cells. A minimum of 100 cells per sample group was analyzed per experiment.

### Invasion Assay

Transwell inserts (membrane pore size, 8 μm; diameter, 6.4 mm; cat. no. 353097, FALCON^®^, Corning Inc., United States) were placed in 24-well plates. The cells were seeded in serum-free medium in upper chambers (150,000 cells per chamber for U87 and U87-TxR; 70,000 cells per chamber for C6 and RC6) covered with a Matrigel layer (500 ng/ml; cat. no. 356234, Basement Membrane Matrix, BD Biosciences, United States) and subsequently treated with 2 μM **5** and 8 μM **6**. The lower chambers were filled with appropriate medium supplemented with 10% fetal bovine serum as a chemoattractant. A negative control without 10% fetal bovine serum was also included in each experiment, as a measurement of spontaneous cell invasion. After 24 h, cells that invaded through the Matrigel and membrane were fixed in 4% paraformaldehyde. Cells remaining on the upper surface of the membrane were carefully removed with a cotton swab. Invading cells from the lower surface of the membranes were stained with Hoechst 33342. Membranes were carefully removed from inserts and placed on microscopic glass slides. Stained cells were counted under a Zeiss Axio Vert inverted fluorescent microscope at 10× magnification. Stained cells were counted in ImageJ software (1.48, Microsoft, Redmond, WA, United States).

### Statistical Analysis

Statistical analyses were performed by GraphPad Prism 6 (GraphPad Software, Inc., San Diego, CA, United States). Data normality was estimated using the D’Agostino and Pearson omnibus normality test. The data obtained by MTT assay, qRT-PCR, and flow cytometric immunostaining were analyzed by two-way ANOVA. Data from gelatin degradation and invasion assays, not having normal distribution, were analyzed by the Kruskal–Wallis test. The observed differences were considered statistically significant if *p* < 0.05.

## Results

### The Six UMAs Inhibit the Viability of Glioma Cells

Firstly, we assessed the effect of the six UMAs on the metabolic activity of viable rat (C6 and RC6) and human (U87 and U87-TxR) glioma cells after 72 h treatment by MTT assay. The results are compared to previously reported data obtained in peripheral blood mononuclear cells (PBMCs) (Jovanovic et al., 2019) and summarized in Table 1. All six compounds expressed significant inhibition of viability in all glioma cell lines. **1** and **2** showed no selectivity toward human glioma cell lines compared to PBMCs. Furthermore, MDR U87-TxR cells were more resistant toward **1** and **2** (2.7- and 1.6-fold, respectively) compared to their corresponding sensitive U87 cells. The other four compounds exhibited selectivity toward glioma cell lines, with the highest selectivity observed after compound **6** treatment. MDR RC6 cells were moderately resistant to **3** and **4** compared to their sensitive counterparts.

**TABLE 1:** Cytotoxicity of UMAs in C6, RC6, U87, and U87-TxR cell lines and PBMCs.

Compound ID	IC_50_ (μM)				
	
	C6	RC6	U87	U87-TxR	PBMC
**1**	1.250.34	1.730.25	1.410.13	3.780.78	1.620.5
**2**	6.430.81	5.641.45	15.191.81	24.1610.81	15.343.80
**3**	0.460.15	0.860.1	0.460.06	0.670.04	2.100.83
**4**	0.820.07	1.320.71	0.800.08	0.610.1	5.600.80
**5**	1.680.27	2.190.57	2.920.34	2.570.35	7.292.59
**6**	10.812.83	8.151.08	9.700.39	9.310.32	55.715.12

*Values are presented as IC_50_ ± SE in μM.*

### The UMAs Modulate Oxidative Stress in Glioma Cell Lines

**FIGURE 1:**
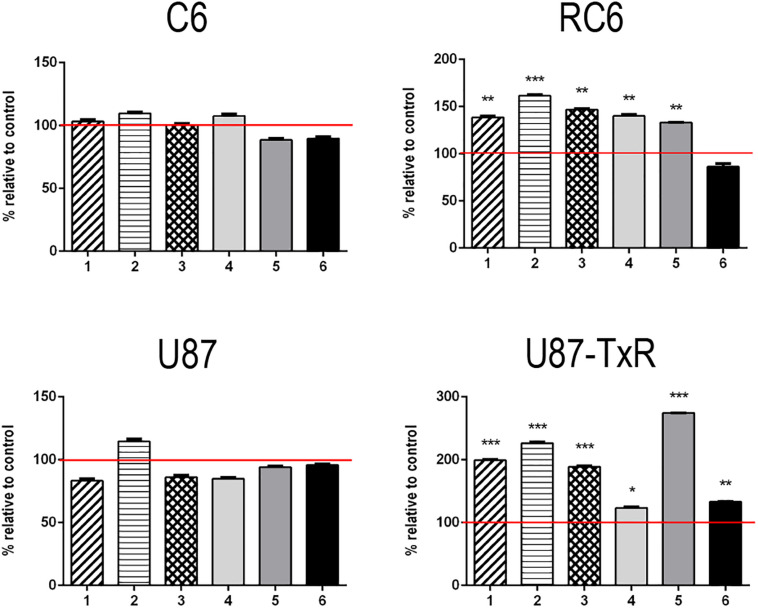
Changes in RONS production induced by **5** and **6**. Detection of the RONS production level was performed by DHR labeling. DHR mean fluorescence intensity in stained cells of C6, RC6, U87, and U87-TxR cell lines, treated with compounds **1**, **2**, **3**, **4**, **5**, and **6** relative to untreated control (set at 100%, red line). All results are presented as percentages normalized to untreated control obtained from three independent experiments (*n* = 3).

To investigate whether inhibition of TrxR1 by UMAs induces oxidative stress in glioma cell lines with different antioxidative capacities (Stankovic et al., 2015; Stojkovic et al., 2015), we assessed the levels of RONS (peroxynitrite anion and hydrogen peroxide) with fluorescent dye DHR by flow cytometry (Supplementary Figure 2). The results of DHR staining upon 24 h treatment with six UMAs are displayed as percentages relative to control untreated cells (Figure 1). **1**–**5** demonstrated an increase in DHR fluorescence in RC6 and U87-TxR cells, with a negligible effect on sensitive cell lines (Figure 1). Changes in the RONS level in U87-TxR cells were more pronounced. The most prominent increase in RONS production (2.7-fold) was induced by compound **5** in U87-TxR cells (Figure 1). Based on selectivity toward cancer cell lines, the same efficacy in sensitive and MDR glioma cell lines, and capacity to increase RONS level, **5** (DVD-444) was chosen for further studies. Compound **6** (DVD-445) was used as a reference UMA particularly studied in our recent publications (Jovanovic et al., 2019, 2020).

### 5 and 6 Induce Changes in mRNA Expression of Antioxidative Enzymes

**FIGURE 2:**
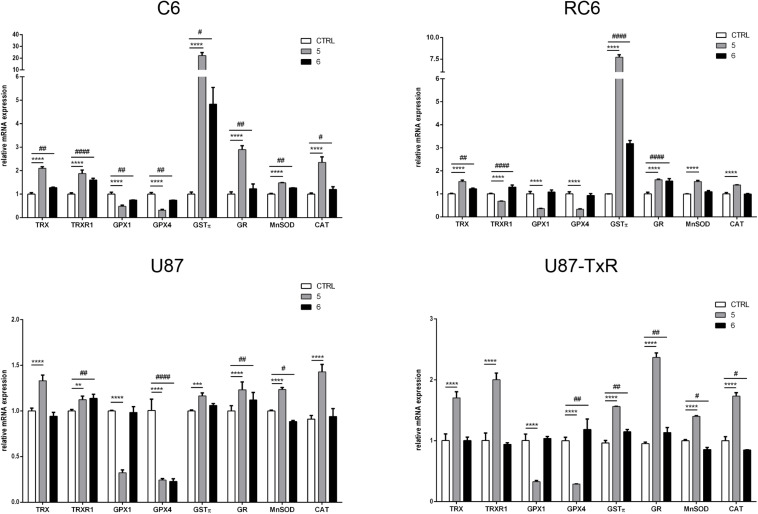
Quantitative real-time PCR analysis of changes in antioxidative enzymes expression in C6, RC6, U87, and U87-TxR cell lines, induced by 2 μM **5** and 8 μM **6**. The mRNA expression of *Trx1*, *TrxR1*, *GPx1*, *GPx4*, *GST*π, *GR*, *MnSOD*, and *CAT* was normalized to *ACTB* as internal control. All results represent mean values ± SD, obtained from three independent experiments (*n* = 3). *p* < 0.01 (**), *p* < 0.001 (***), and *p* < 0.0001 (****) indicate significantly different level of expression in cells treated with **5** in comparison with untreated control. *p* < 0.05 (#), *p* < 0.01 (##), and *p* < 0.0001 (####) indicate statistical significance between untreated control and cells treated with **6**.

Next, we analyzed the mRNA expression levels of enzymes involved in maintaining redox balance by qRT-PCR after 24 h treatment with TrxR1 inhibitors (Figure 2). Components of the Trx system (Trx and TrxR1), GSH detoxification system (GPx1, GPx4, GSTπ, and GR), and antioxidant enzymes MnSOD and CAT were investigated. As expected, both TrxR1 inhibitors caused an increase in mRNA expression of *TRX* and *TRXR1*. However, *TRX* and *TRXR1* mRNA expression levels remained unchanged upon treatment with compound **7** in the U87-TxR cell line (Figure 2). Interestingly, **5** significantly decreased the expression of *GPx1* and *GPx4* mRNA in all four cell lines, while **6** showed a variable effect on *GPx1* and *GPx4* mRNA expressions (Figure 2). The highest increase in *GST*π mRNA expression was observed upon treatment with **5** in C6 and RC6 cells (23- and 7.7-fold, respectively). Treatment with **6** also significantly increased the expression of *GST*π mRNA in these cell lines (4.5- and 3-fold, respectively). Both TrxR1 inhibitors induced expression of *GST*π mRNA, but to a lesser extent in human glioblastoma cell lines (Figure 2). As shown in Figure 2, all glioma cell lines significantly increased the expression of *GR* mRNA after treatment with **5** and **6**. The most pronounced increase was detected with **5** in C6 and U87-TxR cells (3- and 2.3-fold, respectively). **5** caused an increase in mRNA expression of antioxidant enzymes *MnSOD* and *CAT* in sensitive and MDR glioma cell lines, while **6** had no significant effect on mRNA expressions of *MnSOD* and *CAT* (Figure 2).

### 5 and 6 Affect TrxR1, ASK1, and RNR Protein Expression Differently in Glioma Cell Lines

**FIGURE 3:**
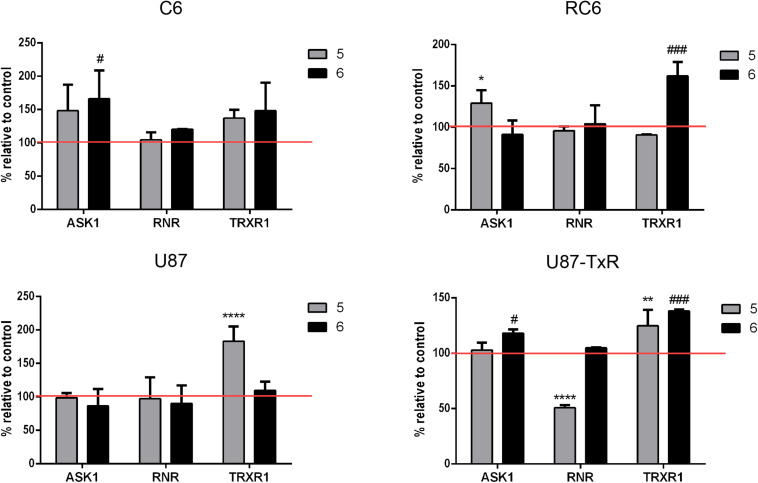
Immunostaining of proteins ASK1, RNR, and TrxR1 in C6, RC6, U87, and U87-TxR cell lines. Changes in expression of proteins in cells treated with 2 μM **5** and 8 μM **6** are compared to untreated control (set at 100%, red line). All results represent mean values ± SD, obtained from three independent experiments (*n* = 3). *p* < 0.05 (*), *p* < 0.01 (**), and *p* < 0.0001 (****) indicate significantly different levels of expression in cells treated with compound **5** in comparison with untreated control. *p* < 0.05 (#) and *p* < 0.001 (###) indicate statistical significance between untreated control and cells treated with **6**.

To investigate whether inhibition of the Trx system cause changes in the expression of downstream proteins, we performed immunostaining with TrxR1-, ASK1-, and RNR-specific antibodies. Following 24 h treatments with **5** and **6**, the samples were analyzed by flow cytometry (Supplementary Figure 3). Results are presented as percentages normalized to control untreated cells (set at 100%) (Figure 3, red lines). **5** increased the expression level of TrxR1 in the C6 cell line, but without statistical significance, while the protein level of TrxR1 remained unchanged in RC6 cells (Figure 3). Protein levels of TrxR1 significantly increased in U87 and U87-TxR cells upon treatment with **5** (Figure 3). **6** caused a significant increase in TrxR1 expression level only in MDR glioma cell lines (Figure 3). Flow cytometric analysis revealed that the expression of ASK1 protein increased following treatment with both TrxR1 inhibitors in the C6 cell line (Figure 3). However, RC6 cells increased the protein level of ASK1 only after treatment with **5**, while U87-TxR cells slightly increased expression of ASK1 protein upon treatment with **6** (Figure 3). The expression of ASK1 protein was not affected following treatment with **5** and **6** in the U87 cell line (Figure 3). Treatment with **5** caused a significant decrease in RNR expression level only in the U87-TxR cell line (Figure 3).

### TrxR1 Inhibitors 5 and 6 Disturb Mitochondrial Membrane Potential and Induce Cell Death in Rat Glioma Cell Lines

**FIGURE 4:**
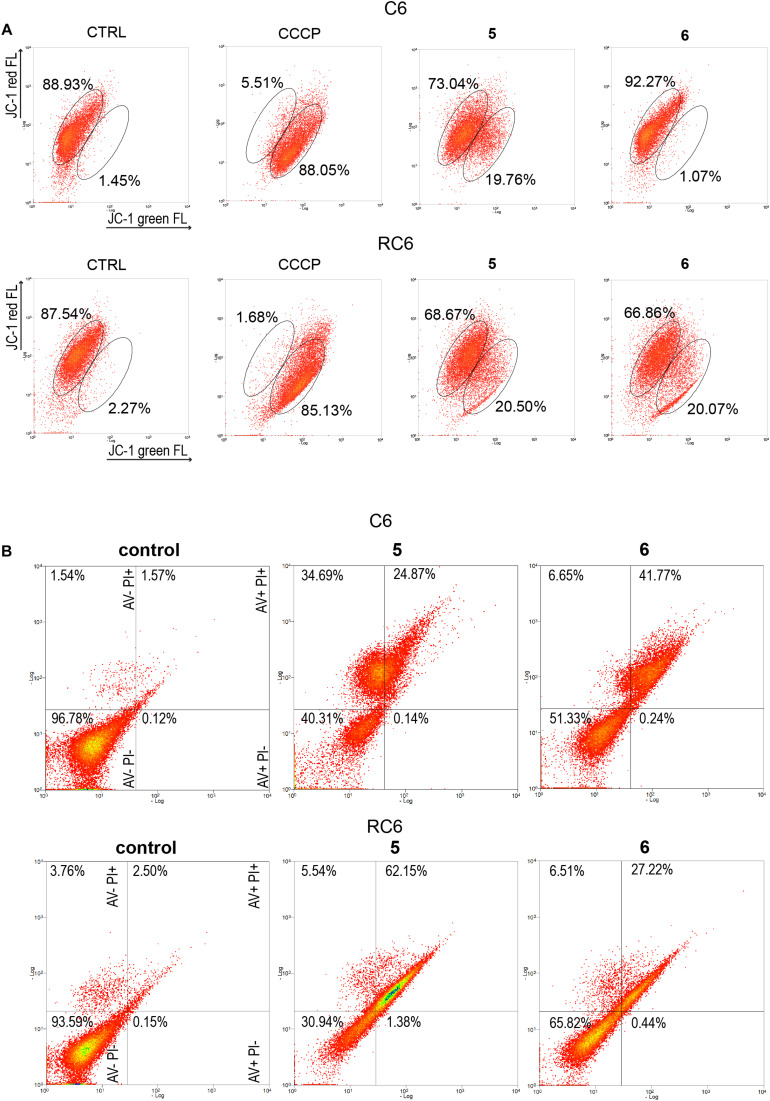
Flow cytometric assessment of mitochondrial membrane depolarization and cell death induction by **5** and **6** in rat glioma cells. **(A)** Flow cytometric profiles of JC1 red and green fluorescence intensity in C6 and RC6 stained cells, treated with 2 μM **5** and 8 μM **6**. Gated areas are selected according to the fluorescence intensity of ≥85% stained cells in untreated (CTRL) cells and CCCP-treated cells as a positive control. Displayed profiles are representative results of three independent experiments (*n* = 3). **(B)** Cell death was assessed by AV/PI staining after 72 h treatment with **5** and **6** in C6 and RC6 cells. Flow cytometric assay distinguishes viable (AV− PI−), early apoptotic (AV + PI−), late apoptotic (AV + PI +), and necrotic (red: AV− PI +) cells. Experiments were performed three times (*n* = 3).

To establish whether **5** and **6** affect mitochondrial function, we analyzed changes in mitochondrial membrane potential after 24 h treatment by JC-1 staining in rat glioma cells (Figure 4A) and human glioblastoma cells (Supplementary Figure 4A). CCCP was used as a positive control due to its significant depolarizing effect on mitochondrial membrane potential. **5** caused a change in mitochondrial membrane potential in all glioma cell lines, with a more prominent depolarization effect in rat glioma cells (Figure 4A and Supplementary Figure 4A). **6** showed the highest potential to change mitochondrial membrane potential in the RC6 cell line (Figure 4A).

To explore how changes in mitochondrial membrane potential and ASK1 protein level caused by TrxR1 inhibitors influence cell death induction, sensitive and MDR glioma cell lines were subjected to AV/PI staining following 72 h treatment with **5** and **6**. Flow cytometric analysis revealed that **5** increased a portion of late apoptotic and necrotic cells, but not early apoptotic cells in the C6 cell line (Figure 4B). Namely, the percentages of late apoptotic and necrotic C6 cells increased from 3.11% (the sum of AV + PI + and AV− PI +) in untreated samples to 59.56% in samples treated with **5**. However, **6** increased only the percentages of late apoptotic C6 cells (from 1.57% in untreated control to 41.77% in treated cells). **5** and **6** significantly increased a portion of late apoptotic RC6 cells (from 2.50% in untreated control to 62.15 and 27.22% in treated cells, respectively). However, in U87 and U87-TxR cell lines, no remarkable induction of cell death was detected upon treatment with TrxR1 inhibitors (Supplementary Figure 4B).

### 5 and 6 Inhibit Cell Proliferation in Human Glioblastoma Cells

**FIGURE 5:**
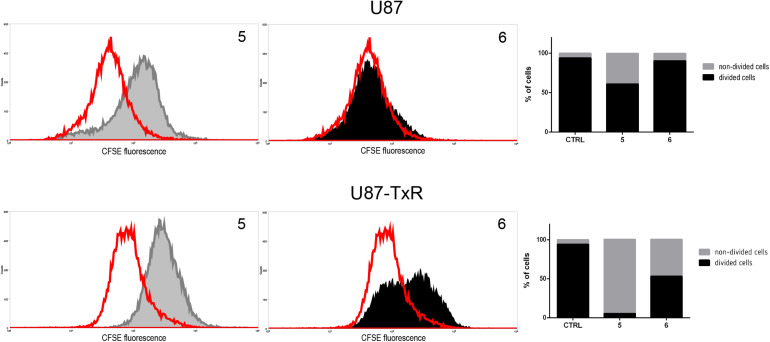
Inhibition of cell proliferation in U87 and U87-TxR cell lines. Flow cytometric profiles of CFSE staining after 72 h treatment with 2 μM **5** and 8 μM **6**. CFSE fluorescence declines following each cell division. Red-line areas indicate controls, while gray and black areas signify treated cells. Experiments were performed three times (*n* = 3).

Since TrxR1 inhibitors failed to induce cell death in human glioblastoma cell lines, we further explored possible causes underlying the effect on cell viability induced by **5** and **6**. Therefore, the effect of TrxR1 inhibitors on U87 and U87-TxR cell proliferation was analyzed using CFSE staining. The intensity of CFSE fluorescent signal declines following each cell division, meaning that the CFSE distribution in the cells can estimate the rate of cell proliferation. **5** demonstrated significant antiproliferative effect in both U87 and U87-TxR cell lines, while **6** inhibited proliferation of U87-TxR cells (Figure 5).

### TrxR1 Inhibitors Synergize With TMZ Chemotherapeutic Effect in Glioma Cells

**FIGURE 6:**
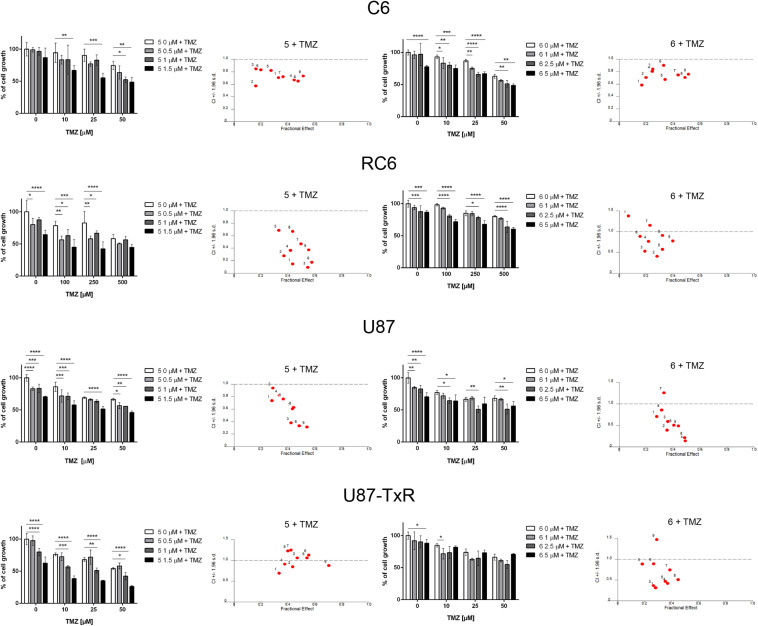
The combined effect of **5** or **6** with TMZ in glioma cell lines. Cell growth inhibition by TMZ in single and combined treatments with **5** and **6** in C6, RC6, U87, and U87-TxR cells determined by MTT assay. Mean values ± SEM were obtained from three independent experiments (*n* = 3). Statistical significance to TMZ alone: *p* < 0.05 (*), *p* < 0.01 (**), *p* < 0.001 (***), and *p* < 0.0001 (****). The interactions between **5** or **6** and TMZ was analyzed by CalcuSyn software. CI < 1 indicates synergistic effect, while CI close to 1 point to additive effect.

The interaction between **5** and **6** and TMZ during combined treatments in glioma cell lines was assessed by MTT assay (Figure 6). These results were subjected to computerized synergism/antagonism CalcuSyn software analysis. The majority of examined combinations between TrxR1 inhibitors and TMZ demonstrated additive (CI values close to 1) or synergistic (CI < 1) interactions (Supplementary Tables 1, 2). Importantly, the compounds potentiated the effect of TMZ in RC6 cells resistant to TMZ as well.

### 5 and 6 Modify P-gp Activity and Sensitize the U87-TxR Cell Line to PTX

**FIGURE 7:**
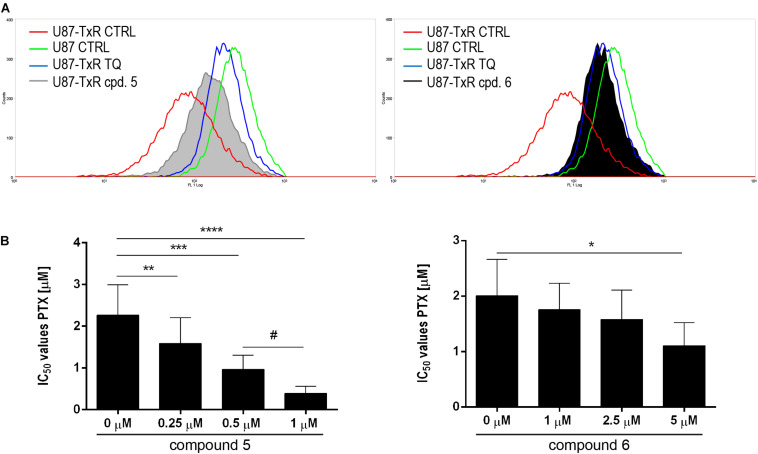
**5** and **6** inhibit P-gp function and enhance the sensitivity of U87-TxR cells to PTX. **(A)** Flow cytometric profile of Rho123 accumulation in untreated U87 and U87-TxR cells and U87-TxR cells treated with 1 μM **5**, 5 μM **6**, and TQ as positive control. **(B)** Relative reversal of PTX resistance in combination with subinhibitory concentrations of **5** and **6** in the U87-TxR cell line. Statistical significance to PTX alone: *p* < 0.05 (*), *p* < 0.01 (**), *p* < 0.001 (***), and *p* < 0.0001 (****), and PTX in combination with 0.5 and 1 μM: *p* < 0.05 (#).

To investigate possible interaction of **5** and **6** with P-gp, intracellular accumulation of Rho123 (a P-gp substrate) was analyzed by flow cytometry in the U87-TxR cell line with P-gp overexpression (Figure 7). The accumulation of Rho123 was compared with TQ, a non-competitive P-gp inhibitor. Flow cytometric profiles of Rho123 accumulation are presented in Figure 7A. One micromolar of compound **5** and 5 μM of **6** induced a marked increase in Rho123 accumulation. Considering the potential of **5** and **6** to modify P-gp function, we examined their potential to sensitize U87-TxR cells to PTX (Figure 7B). The effects of the simultaneous combination of TrxR1 inhibitors with PTX were assessed by the MTT assay after 72 h treatment. Both compounds applied at subinhibitory concentrations sensitized U87-TxR cells to PTX. **5** decreased the IC_50_ values for PTX in a concentration-dependent manner, while all applied concentrations of **6** showed similar potential to reverse PTX resistance. Moreover, the sensitizing effect was more prominent with **5**.

### 5 and 6 Suppress the Invasive Potential of Glioma Cells

**FIGURE 8:**
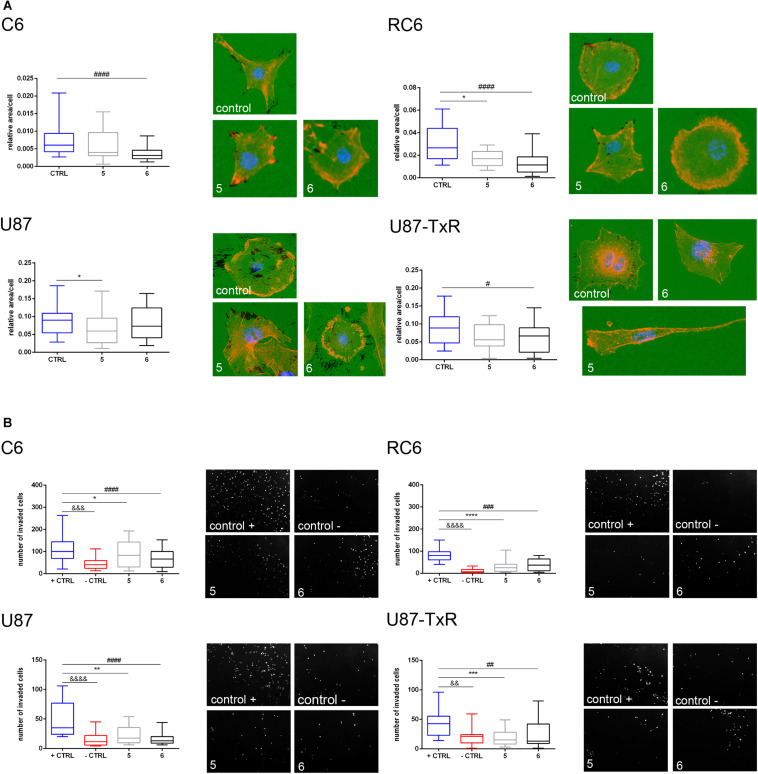
Assessment of migratory and invasive potential of glioma cell lines upon treatment with **5** and **6**. **(A)** Representative images of gelatin degradation by C6, RC6, U87, and U87-TxR cells after 24 h treatment with 2 M m **5** and 8 μM **6** are attributed to box plots that show a degraded surface of gelatin per total area of cells in control and treatments. **(B)** Representative images of C6, RC6, U87, and U87-TxR cells that migrated through Matrigel and passed the insert membrane pores obtained after 24 h treatment with 2 μM **5** and 8 μM **6**. Box plots show the number of cells that invaded through the Matrigel to the opposite side of the membrane. Control + is positive control (with FBS as chemoattractant) and control- is negative control (without chemoattractant). All results are obtained from at least three independent experiments. *p* < 0.05 (*), *p* < 0.01 (**), *p* < 0.001 (***), and *p* < 0.0001 (****) indicate statistical significance of **5**-treated cells compared to untreated control. *p* < 0.05 (#), *p* < 0.01 (##), *p* < 0.001 (###), and *p* < 0.0001 (####) indicate statistical significance between cells treated with **6** and untreated control. *p* < 0.01 (&&), *p* < 0.001 (&&&), and *p* < 0.0001 (&&&&) indicate statistical significance between negative and positive controls.

Next, we wanted to examine whether **5** and **6** have the potential to inhibit the migratory and invasive properties of glioma cells. Gelatin degradation assay was performed to assess the potential of glioma cells to degrade the extracellular matrix. Results, obtained after 24 h treatment with **5** and **6**, are summarized in Figure 8A. Compound **6** significantly decreased the potential of C6, RC6, and U87-TxR cells to degrade gelatin (Figure 8A). Compound **5** suppressed gelatin degradation in MDR rat cell lines and human sensitive cell lines (Figure 8A).

Furthermore, we performed an invasion assay to explore the potential of glioma cells to invade through the basement membrane after 24 h treatment with **5** and **6**. As presented in Figure 8B, both compounds significantly decreased the potential of glioma cells to invade through the basement membrane. Importantly, both compounds showed a higher anti-invasive effect in MDR glioma cell lines (Figure 8B).

## Discussion

The main reasons for inefficient GBM treatment are elevated intratumor and intertumor heterogeneity, drug resistance, and dissemination of GBM cells into surrounding brain tissues. Altered expression of antioxidant enzymes is a significant feature of GBM heterogeneity (Dokic et al., 2012). Moreover, redox systems play an important role in GBM progression, metastasis, and resistance to therapy. Consequently, developing novel therapeutics targeting antioxidant capacity presents a promising strategy for GBM treatment. Herein, we report novel TrxR1 inhibitors able to induce oxidative stress in glioma cells. These inhibitors differently affected rat and human glioma cells by inducing cell death and by suppressing cell proliferation. Importantly, novel TrxR1 inhibitors were capable of sensitizing glioma cells to chemotherapy and of decreasing the invasion of glioma cells.

The Trx system is one of the major defense systems against redox imbalance. Therefore, in the present study, we evaluated the potential of novel TrxR1 inhibitors to induce oxidative stress in two pairs of sensitive and MDR glioma cell lines with different antioxidative capacities. The rat glioma RC6 cell line was selected from C6 cells by continuous exposure to increasing concentrations of a GBM approved drug—carmustine. RC6 cells also showed cross-resistance to TMZ. The main mechanism of resistance in RC6 cells is an adaptation to oxidative stress with an elevated level of RONS production and higher expression of antioxidant enzymes (GPx1, MnSOD, and iNOS) (Stojkovic et al., 2015). The human MDR glioblastoma U87-TxR cell line was established from the U87 cell line after continuous exposure to PTX. The main characteristics of U87-TxR cells are overexpression of P-gp and lower RONS production, accompanied by lower GSH content and GSTπ expression (Podolski-Renic et al., 2011). We hypothesized that differences in the antioxidative potential of these two MDR glioma models would have an impact on their capacities to respond to oxidative stress induced by TrxR1 inhibitors. However, based on RONS production, expression of antioxidant enzymes, and perturbation of mitochondrial membrane potential, we observed the novel TrxR1 inhibitors were able to induce oxidative stress regardless of antioxidative potential in MDR glioma cells.

Among six tested UMAs–TrxR1 inhibitors, which showed a potent effect on glioma cell viability, we chose **5** due to the same efficacy in sensitive and corresponding MDR cells. Most tested TrxR1 inhibitors were less effective in PBMCs showing selectivity toward cancer cells, which is in line with previous studies (Karlenius and Tonissen, 2010; Zhang et al., 2017). Compound **6** that we studied as one of the most potent TrxR1 inhibitor (Jovanovic et al., 2019, 2020) was used as a reference UMA for in-depth study of **5** mechanisms of action in glioma cells.

Inhibition of TrxR1, and consequentially the whole Trx system, leads to an increase in oxidative stress in the cell (Arner, 2009; Galadari et al., 2017). To determine whether our inhibitors induce oxidative stress in glioma cell lines, we analyzed changes in the RONS level upon 24 h treatments. Significant elevation of the RONS level was observed only in MDR glioma cells. As expected, U87-TxR cells with low antioxidative potential were the most prone to an increase in the RONS level. **5** showed the highest capacity to induce RONS production.

Given the dynamic nature of RONS and other reactive species in cell milieu, an increase in RONS subsequently activates signaling pathways regulating antioxidant enzymes’ expression. Therefore, an indication of oxidative stress inside of the cell could be the change in the expression of enzymes relevant for maintaining redox balance. **5** and **6** induce significant changes in the expression of antioxidant enzymes in all cell lines, implying the existence of oxidative stress inside the cells. Expectedly, both TrxR1 inhibitors caused an increase in mRNA expression of *TRX* and *TRXR1*, considering cells are trying to compensate for the lack of inhibited enzyme’s function. An increase in TrxR1 expression was further confirmed with TrxR1 immunostaining.

Apart from the Trx system, we also examined the expression of enzymes of the GSH system, another important redox system inside the cell. These two redox systems are connected to maintaining redox status. Hence, perturbation of one redox system—GSH or Trx—is likely to affect another.

Treatments with **5** and **6** caused a significant decrease in mRNA expression of both *GPx1* and *GPx4* in all four glioma cell lines. GPxs are antioxidant enzymes that neutralize peroxides and eliminate peroxynitrite to prevent oxidative damage (Sies et al., 1997). GPx1 is the most abundant enzyme in the family, and it is considered as one of the main enzymes for hydrogen peroxide removal, while GPx4 protects the cell from lipid peroxidation (Brigelius-Flohe, 1999). Decreased expression of GPx1 in brain tumor cells leads to increased sensitivity to oxidative stress (Dokic et al., 2012), while inhibition of GPx4 leads to the accumulation of lipid hydroperoxides and ferroptotic cell death (Seiler et al., 2008; Yang et al., 2014). A decrease of GPxs could significantly contribute to observed cytotoxicity of tested compounds. It has been shown that dual suppression in the activity of both enzymes of the Trx system and GSH system is more effective in cancer cell death induction than inhibition of one of the systems alone (Leone et al., 2017; Wang et al., 2019).

Treatment of glioma cells with Michael acceptors–TrxR1 inhibitors **5** and **6** induced a significant increase in GSTπ expression, presumably as a defense mechanism against applied xenobiotics. Namely, GSTs are upregulated by electrophiles (such as Michael acceptors) through the binding of the Nrf2 transcription factor to the antioxidant response element (ARE sequence, also called electrophile response element) in the promoter region of the GSTs genes. It is also proposed that GSTs contribute to antioxidant defense by catalyzing the reduction of organic peroxides with GSH (Lu, 2009). This could be another reason for the considerable increase in GSTπ expression.

The observed increase in GR upon **5** and **6** treatments could be the consequence of cell stress imposed by treatment with TrxR1 inhibitors. To maintain the redox potential of the GSH system, cells need to be more efficient in the reduction of accumulated GSSG. GR is an enzyme responsible for GSSG reduction to GSH. Moreover, GSH is an efficient scavenger of peroxynitrite and plays a major role in the cellular defense against this species (Pacher et al., 2007).

**5** caused an increase in mRNA expression of *MnSOD* and *CAT* in sensitive and MDR glioma cell lines. MnSOD conjugates superoxide anion to hydrogen peroxide. As the first line of mitochondrial defense mechanisms against oxidative damage, changes in the expression of this enzyme reflect a change in mitochondrial oxidative stress (Kim, 2010). As previously mentioned, CAT corresponds in function with GPx and neutralizes hydrogen peroxide (Birben et al., 2012). Nonetheless, Dokic et al. (2012) reported that CAT cannot compensate in function for the significantly decreased expression of GPx1. Therefore, the oxidative stress followed by GPx1 decrease induced by **5** treatment cannot be overcome with the increase in CAT expression.

The increase in mitochondrial membrane potential caused by **5** corresponds to the increase in *MnSOD* expression in RC6 cells. Changes in expression of antioxidant enzymes, together with perturbed mitochondrial membrane potential, are strong indications of oxidative stress inside of the cell.

Inhibition of TrxR, followed by increased oxidative stress and disrupted mitochondrial membrane potential, ultimately leads to cell death (Arner, 2009; Galadari et al., 2017). Indeed, **5** and **6** induced late apoptosis and necrosis in rat glioma cell lines. Inhibition of the Trx system with **5** and **6** provoked an increase in ASK1 protein content in C6 and RC6 cell lines, which is in agreement with the observed late apoptosis in these cell lines upon application of TrxR1 inhibitors. Namely, reduced Trx inhibits the function of ASK1, thus preventing apoptosis (Saitoh et al., 1998). Also, binding of reduced Trx to ASK1 targets ASK1 for ubiquitin-mediated degradation (Liu and Min, 2002). In human glioblastoma cell lines, we did not observe significant induction of cell death despite evident effect on cell viability upon treatment with TrxR1 inhibitors. However, the protein content of RNR was significantly decreased by **5** treatment in U87-TxR cells, implying that synthesis of deoxyribonucleotides could be affected. Knowing that Trx, as a proton provider, enables RNR to perform the synthesis of deoxyribonucleotides (Lu and Holmgren, 2014), we investigated the antiproliferative effect of TrxR1 inhibitors in U87 and U87-TxR cells. Indeed, TrxR1 inhibitors suppressed proliferation in both human glioblastoma cell lines. However, the inhibition of cell proliferation was more pronounced in U87-TxR cells.

As previously mentioned, first-line chemotherapy against GBM is TMZ. It is an alkylating agent that induces DNA damage and has good penetrability through the BBB (Ostermann et al., 2004). As a defense mechanism against TMZ, glioma cells boost up antioxidant systems, displaying higher content of GSH and GR. On the other hand, suppression of the GSH system sensitizes cells to TMZ (Zhu et al., 2018). Different redox modulatory compounds are being investigated in combination with TMZ, in order to improve the effect of TMZ. Some of these compounds are even unspecific Trx system inhibitors (Salazar-Ramiro et al., 2016). Our results with **5** and **6** further support the hypothesis that inhibitors of redox regulators have a synergistic effect with TMZ and are suitable for combined treatments with the chemotherapeutic on glioma cells.

P-gp overexpression is one of the major features of the MDR phenotype (Eckford and Sharom, 2009). Furthermore, BBB abundantly expresses P-gp which protects the brain from xenobiotics (Bartels, 2011). To investigate whether TrxR1 inhibitors change P-gp function, we analyzed the accumulation of fluorescent P-gp substrate Rho123 in U87-TxR cells. Our results proved that **5** and **6** directly interact with P-gp, in concentrations of 1 and 5 μM, respectively, as evidenced by increased Rho123 accumulation after 30 min. Due to the similar IC_50_ values obtained in U87 and U87-TxR cells by MTT assay, **5** and **6** cannot be considered as substrates for P-gp. These compounds probably act as P-gp inhibitors, thus possessing the potential to modulate MDR as confirmed by simultaneously combining **5** and **6** with PTX (a chemotherapeutic extruded by P-gp). According to our results, TrxR1 inhibitors sensitized U87-TxR cells to PTX even when applied in sub-efficient concentrations (below their IC_50_ values).

Necrosis is one of the hallmarks of GBM, with necrotic tumor tissue surrounded by highly invasive cells. To survive, GBM cells need to migrate away from the necrotic area. Diffused infiltration into surrounding brain tissues is a limiting factor of curative resection and an efficient way of GBM cells to evade radiotherapy and chemotherapy as well. Both redox systems, GSH and Trx, have an active role in tumor metastasis and progression (Harris et al., 2015). Trx1 increases cancer cell motility and invasion (Bhatia et al., 2016). Trx promotes the activity of matrix metalloproteinases 2 and 9 (Farina et al., 2001), overexpressed by many cancers and highly implicated in acquiring and maintaining metastatic phenotype. We demonstrated that compound **6** significantly inhibited gel degradation both in MDR glioma cells and in the C6 cell line. Both compounds displayed significant inhibition of glioma invasion through Matrigel. Importantly, both compounds showed a higher anti-invasive effect in MDR glioma cell lines. Our results were in agreement with previous findings, where specific TrxR inhibitors ethaselen and auranofin suppressed migration and invasion of breast cancer cells (Bhatia et al., 2016; Zheng et al., 2019).

In conclusion, we found that novel TrxR1 inhibitors induced oxidative stress, leading to disruption of mitochondrial membrane potential and changes in the expression of antioxidant enzymes. Consequently, these inhibitors affected cell proliferation, induced cell death, and suppressed invasion in glioma cells. Importantly, TrxR1 inhibitors were able to modulate redox balance in RC6 glioma cells adapted to oxidative stress and with increased antioxidative capacity. Moreover, TrxR1 inhibitors were capable of overcoming MDR and synergistically interacting with TMZ in glioma cells. Considering the fact that drug resistance and invasion are the main causes of ineffective GBM treatment, UMAs-TrxR1 inhibitors, particularly **5**, could be valuable candidates for the new GBM treatment strategy.

## Data Availability Statement

All datasets presented in this study are included in the article/Supplementary Material.

## Author Contributions

MP and AP-R conceived the study. MK donated the tested compounds. DZ and DD synthetized the tested compounds. MJ and MD performed the experiments. MJ analyzed and interpreted the data. MJ and AP-R drafted the manuscript. MP and MK reviewed and edited the manuscript. All authors read and approved the final manuscript.

## Conflict of Interest

The authors declare that the research was conducted in the absence of any commercial or financial relationships that could be construed as a potential conflict of interest.

## Abbreviations

AV/PI: annexin V/propidium iodide
ARE: antioxidant response element
ASK1: apoptosis signal-regulating kinase 1
ACTB: β-actin
BBB: blood–brain barrier
CAT: catalase
CCCP: carbonyl cyanide *m*-chlorophenyl hydrazine
CFSE: carboxyfluorescein succinimidyl ester
DHR: dihydrorhodamine 123
GBM: glioblastoma
GSH: glutathione
GST: glutathione-S transferases
GPx: glutathione peroxidases
GR: glutathione reductase
MDR: multidrug resistance
NRF2: nuclear factor erythroid 2-related factor 2
P-gp: P-glycoprotein
PTX: paclitaxel
PBMCs: peripheral blood mononuclear cells
PBS: phosphate buffer solution
RONS: reactive oxygen and nitrogen species
Rho123: rhodamine 123
RNR: ribonucleotide reductase
SOD: superoxide dismutase
TMZ: temozolomide
Trx: thioredoxin
TrxR: thioredoxin reductase
UMAs: Ugi-type Michael acceptors.
