# Supplementary material for: Novel TrxR1 Inhibitors Show Potential for Glioma Treatment by Suppressing the Invasion and Sensitizing Glioma Cells to Chemotherapy
Source: Front Mol Biosci. 2020 Oct 6;7:586146. doi: 10.3389/fmolb.2020.586146 (PMC7573255; doi:10.3389/fmolb.2020.586146)
Supplement: Supplementary file 1 [file Data_Sheet_1.PDF]

Supplementary Material

Supplementary Figures

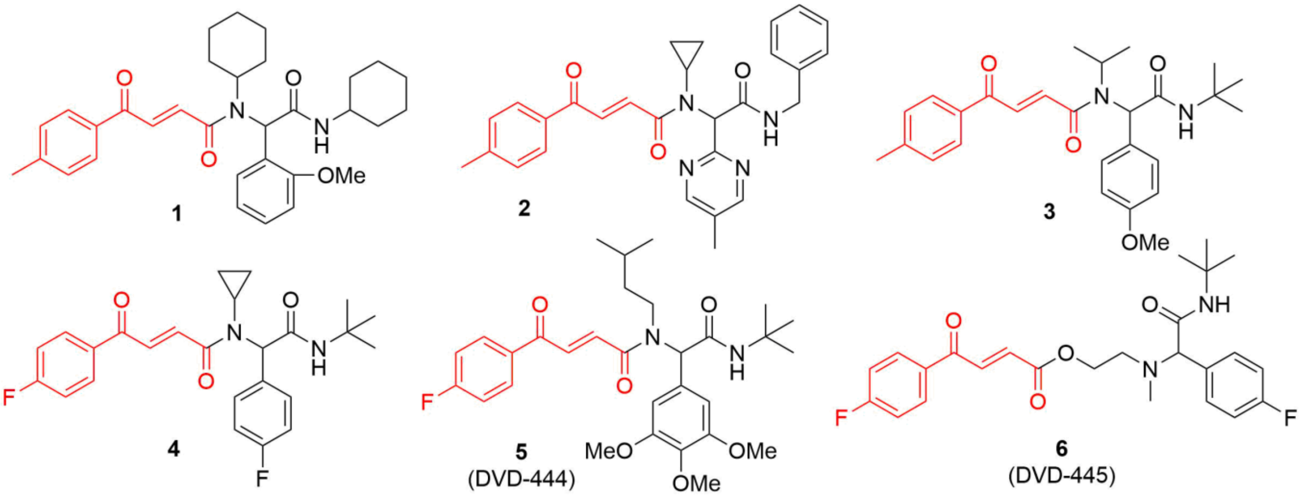

Supplementary Figure 1. Structures of Ugi-type Michael Acceptors.

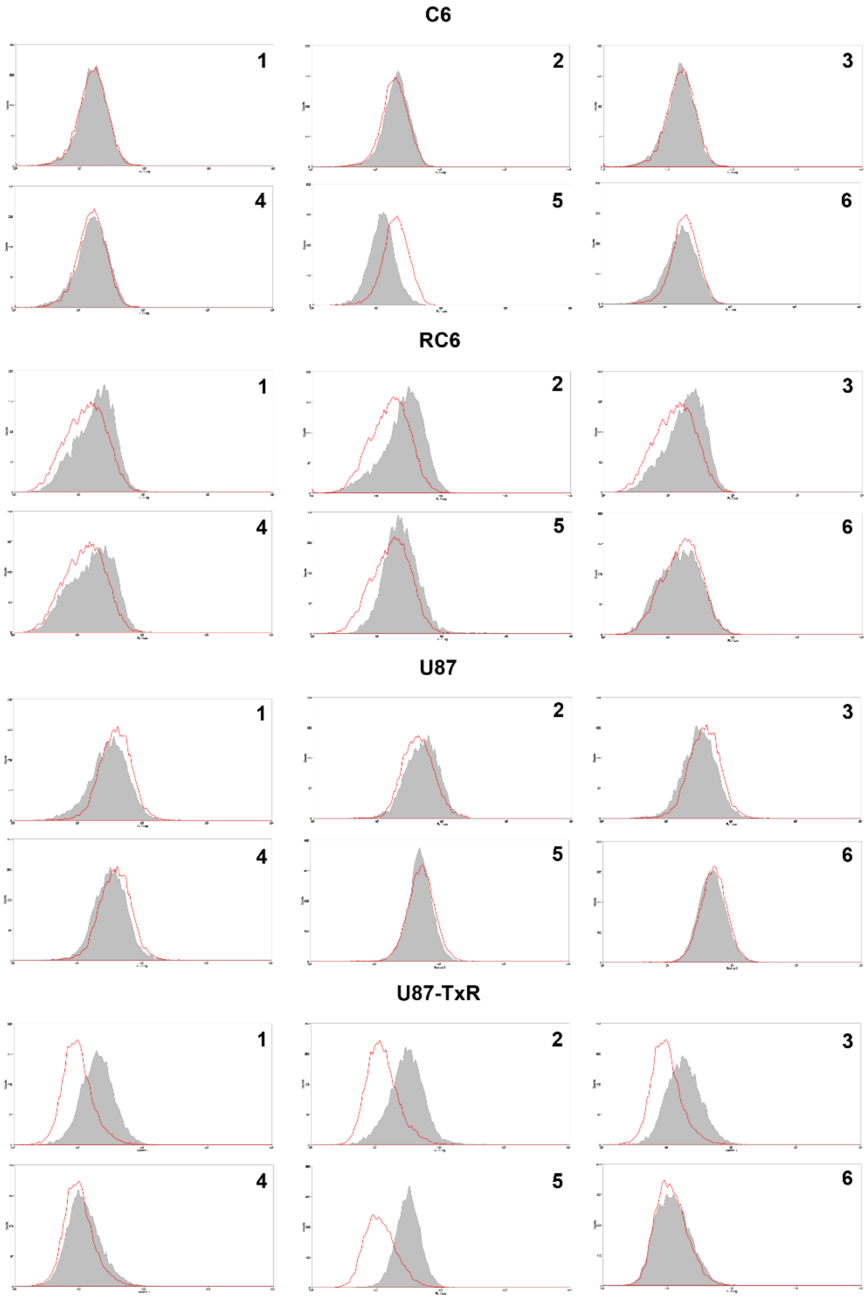

Supplementary Figure 2. Flow cytometric profiles of DHR fluorescence intensity in stained cells of C6, RC6, U87, U87-TxR cell lines, treated with compounds **1**, **2**, **3**, **4**, **5,** and **6** (grey areas) relative to untreated control (red line). The cells were treated for 24 h with 1.5 µM **1**, 5 µM **2**, 0.5 µM **3**, 0.8 µM **4**, 2 µM **5** and 8 µM **6** for rat glioma cells, and 2 µM **1**, 15 µM **2**, 0.5 µM **3**, 0.6 µM **4**, 2 µM **5** and 8 µM **6** for human glioblastoma cells Representative profiles are displayed out of three independent experiments (n = 3).

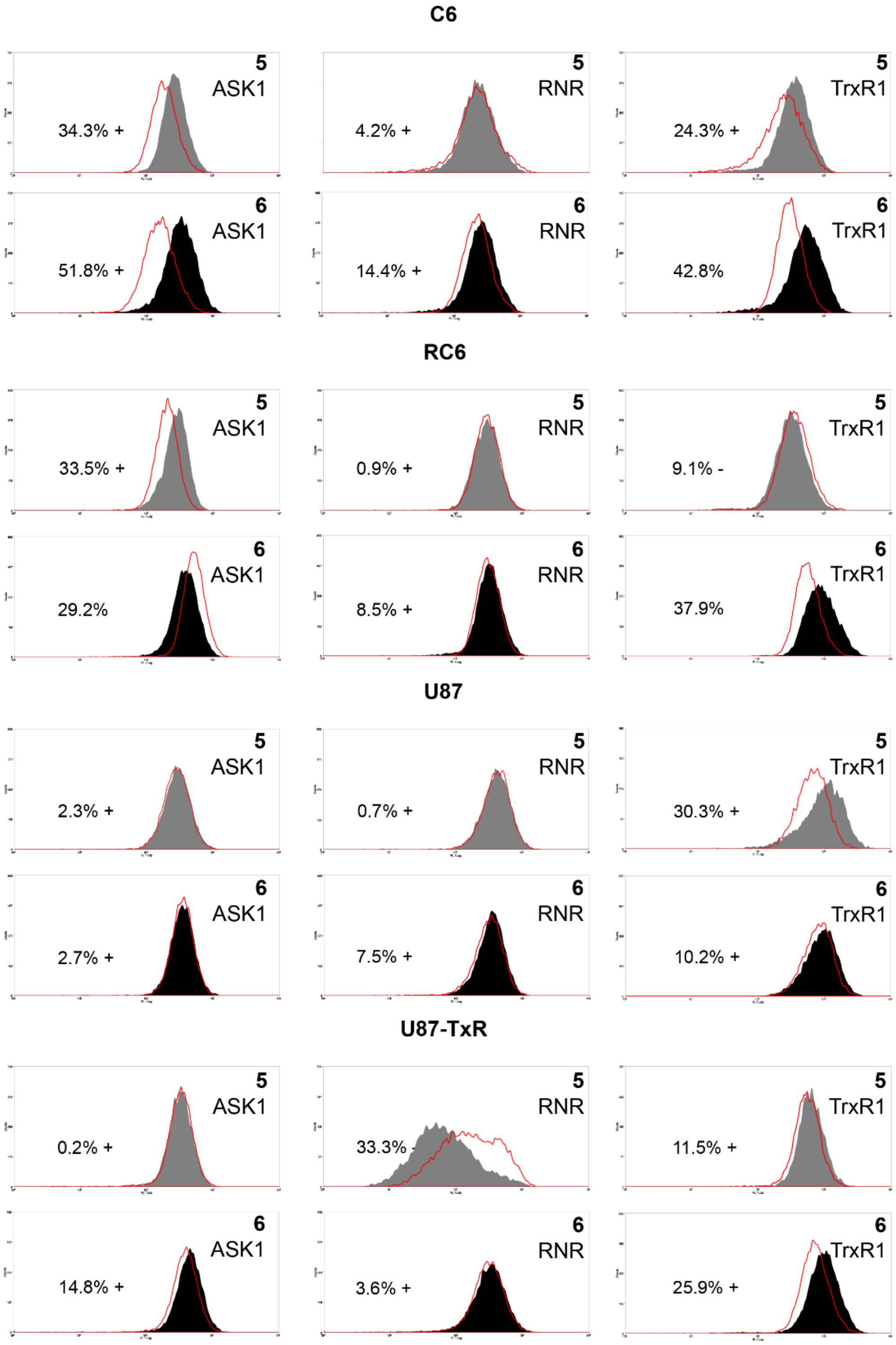

Supplementary Figure 3. Flow cytometric profiles of fluorescently labelled ASK1, RNR, and TrxR1 proteins in C6, RC6, U87, and U87-TxR cell lines. Change in the fluorescence intensity in cells treated with 2 μM **5** (grey areas) or 8 μM **6** (black areas) are compared to untreated control (red lines). Percentages refer to increase (+) or decrease (-) in the fluorescence intensity in treated samples relative to control.

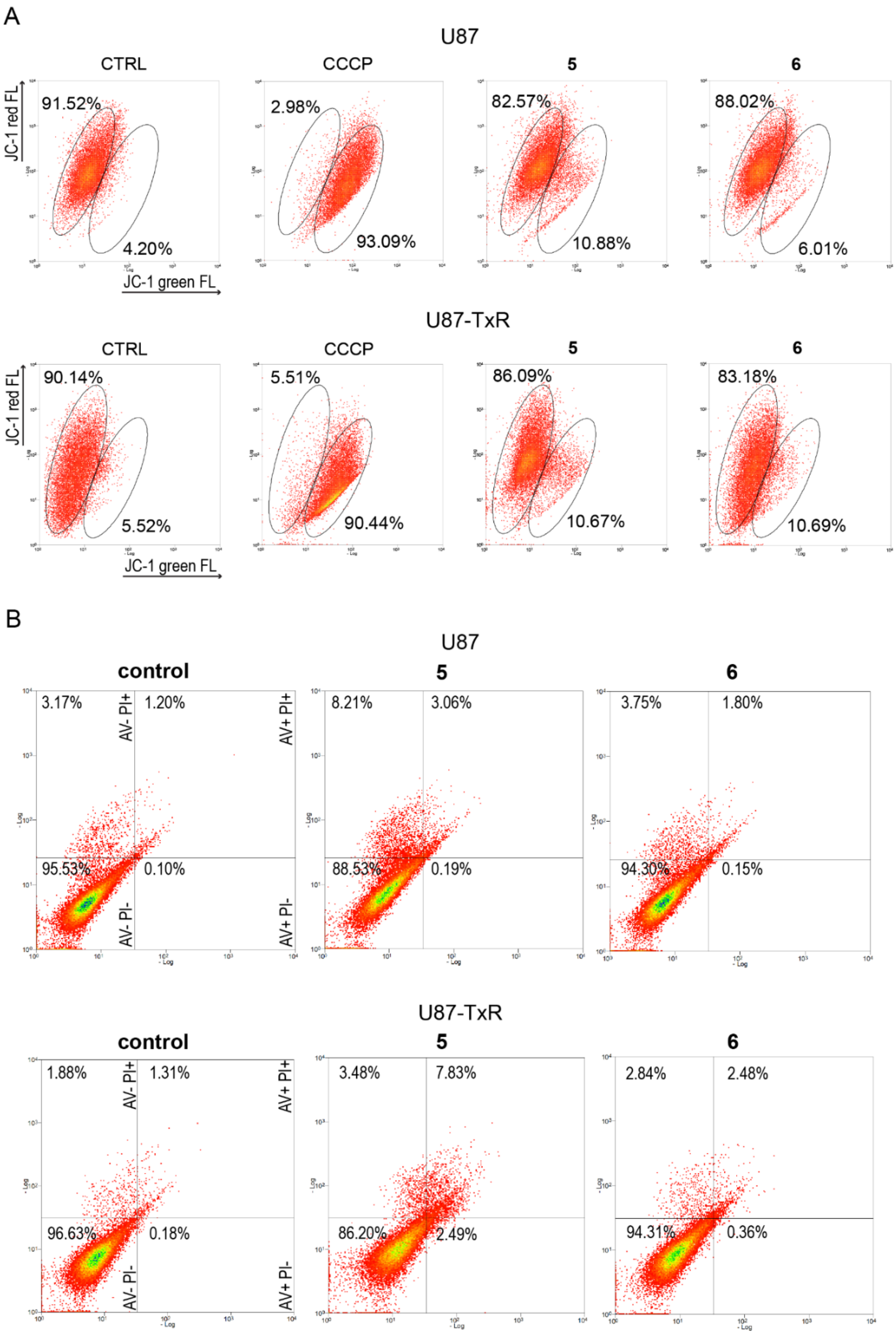

Supplementary Figure 4. Flow cytometric assessment of mitochondrial depolarization by JC-1 staining (A) and cell death induction by AV/PI staining (B) after **5** and **6** treatment in human glioma

cells. **A.** Flow cytometric profiles of JC1 red and green fluorescence intensity in U87 and U87-TxR stained cells, treated with 2 μM **5** and 8 μM **6**. Gated areas are selected according to fluorescence intensity of ≥ 85% of stained cells in untreated (left) and positive control (right). Displayed profiles are representative results of three independent experiments (n = 3). **B.** Cell death was assessed by AV/PI staining after 72 h treatment with **5** and **6** in U87 and U87-TxR cells. Flow cytometric assay distinguishes viable (AV- PI-), early apoptotic (AV+ PI-), late apoptotic (AV+ PI+), and necrotic (AV- PI+) cells. Experiments were performed three times (n = 3).

Supplementary Table 1. Combination index (CI) values for the interaction between TrxR1 inhibitors and TMZ in C6, U87 and U87-TxR cells. Interaction between drugs was determined by CI calculation. CI < 1 indicates synergism, CI = 1 indicates an additive effect, CI > 1 point to antagonism.

**TMZ, [μM]**	**5, [μM]**	**CI 5 vs. TMZ**			**TMZ, [μM]**	**6, [μM]**	**CI 6 vs. TMZ**		
		C6	U87	U87-TxR			C6	U87	U87-TxR
**10**	**0.5**	0.574	0.735	0.690	**10**	**1**	0.582	0.708	0.312
	**1**	0.847	0.939	0.844		**2.5**	0.706	0.392	0.368
	**1.5**	0.705	0.380	1.060		**5**	0.840	0.593	0.888
**25**	**0.5**	0.832	0.820	0.911	**25**	**1**	0.808	0.858	0.421
	**1**	0.822	0.764	1.225		**2.5**	0.677	0.143	0.472
	**1.5**	0.672	0.333	1.061		**5**	0.901	0.505	0.892
**50**	**0.5**	0.720	0.605	1.247	**50**	**1**	0.749	1.255	0.748
	**1**	0.653	0.630	1.126		**2.5**	0.705	0.217	0.511
	**1.5**	0.734	0.312	0.873		**5**	0.759	0.489	1.472

Supplementary Table 2. Combination index (CI) values for the interaction between TrxR1 inhibitors and TMZ in RC6. Interaction between drugs was determined by CI calculation. CI < 1 indicate synergism, CI = 1 indicates an additive effect, CI > 1 point to antagonism.

**TMZ, [μM]**	**5, [μM]**	**CI, 5 vs. TMZ**	**TMZ, [μM]**	**6, [μM]**	**CI, 5 vs. TMZ**
**100**	**0.5**	0.15	**100**	**1**	1.376
	**1**	0.278		**2.5**	0.525
	**1.5**	0.095		**5**	0.408
**250**	**0.5**	0.366	**250**	**1**	0.884
	**1**	0.686		**2.5**	0.766
	**1.5**	0.174		**5**	0.575
**500**	**0.5**	0.473	**500**	**1**	1.152
	**1**	0.669		**2.5**	0.907
	**1.5**	0.373		**5**	0.776
